# A Comparison of Dilute Aqueous Isethionic Acid and Sulfuric Acid in Hydrolysis of Three Different Untreated Lignocellulosic Biomass Varieties

**DOI:** 10.1021/acs.iecr.3c02314

**Published:** 2023-11-02

**Authors:** Ananda S. Amarasekara, Victor C. Nwankwo

**Affiliations:** † Department of Chemistry Prairie View A&M University, 700 University Drive, Prairie View, Texas 77446, United States; ‡ Center for Energy and Environmental Sustainability Prairie View A&M University, 700 University Drive, Prairie View, Texas 77446, United States

## Abstract

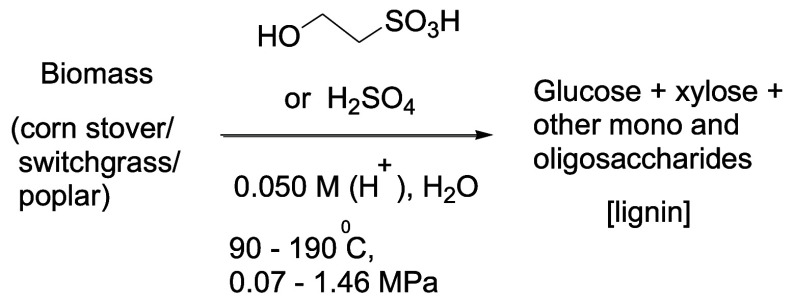

Efficient catalytic hydrolysis of lignocellulosic biomass to sugars is a major challenge in the production of sustainable biofuels and chemical feedstocks. In this study isethionic acid was compared with H_2_SO_4_ for hydrolysis of polysaccharides in corn stover, switch grass, and poplar. The catalytic activities of acids were compared by analysis of total reducing sugar (TRS) and glucose yields in a sequence of experiments in water at 90–190 °C using 0.050 mol of H^+^/L isethionic acid and H_2_SO_4_. In comparison to using H_2_SO_4_, the use of isethionic acid catalyst lowered the maximum TRS percent yield temperatures by 25, 24, and 21% for corn stover, switch grass, and poplar. A similar effect was observed for glucose percent yields as well. This temperature reduction is due to lowering of the activation energy in the polysaccharide depolymerization reaction and most likely due to hydrogen-bonding-type dipolar interactions between the isethionic acid −OH group and −OH groups in biomass polysaccharides.

## 1 Introduction

The anticipated transition from a mostly petroleum-based economy to a renewable energy source and sustainable biomass based economy requires the generation of a large range of bioproducts and biofuels from a vast variety of biomass sources within a flexible and integrated biorefinery. In the last two decades a number of important advancements have been achieved in reaching this goal in areas such as bioethanol, biodiesel, biogas, and renewable resources based polymers.^1^ However, efficient depolymerization of polysaccharides in lignocellulosic biomass to sugars and then processing to fuel and polymer feedstocks is a major challenge in achieving a sustainable carbon based future.^2,3^ The complex molecular architecture of lignocellulosic biomass is composed of cellulose, hemicellulose, and lignin. Cellulose is a β(1–4) linked polymer of d-glucose, whereas hemicellulose is a branched heteropolymer mainly composed of xylan, manan, glucan, and xyloglucans.

The polymeric molecular architecture in lignocellulosic biomass with close packing via numerous strong, inter- and intramolecular hydrogen bonding make it extremely difficult for solvent molecules to break through the arrangement. As a result of this molecular architecture, it is hard to dissolve lignocellulosic biomass in water and in most common organic and water based solvents.^4−6^ High temperature and pressure hydrolysis of the polysaccharide fraction using dilute aqueous sulfuric acid was the classical method used in early cellulosic ethanol plants as far back as 1940s.^2^ This acid catalyzed saccharification gives a sugar solution with glucose, xylose, other monosaccharides, and their oligomers. However, the main disadvantage in dilute sulfuric acid hydrolysis is the poor sugar yield, which affected the ethanol yield. The other disadvantages are high energy cost and high-pressure reactor requirement, while operating at temperatures above 200 °C. With the advancement of enzyme technologies in the last 25 years, the acid hydrolysis process has progressively been replaced by enzymatic hydrolysis.^7^ However, in enzymatic methods, an energy intensive pretreatment is required to facilitate the access of the enzyme to polysaccharides in biomass. Common pretreatments are acid, steam, base, and biochemical methods. The choice of a pretreatment method depends on the chemical composition and physical and textural properties of the lignocellulosic biomass as well as the extent of changes required for a particular enzyme or a mixture of enzymes.^8−10^

In addition, the cost of currently available enzyme preparations and the inability to recycle the enzyme are also major obstacles in economical production of cellulosic ethanol and other cellulose based industrial processes that require depolymerization of cellulose. There are numerous attempts to overcome this challenge and develop a cellulose hydrolysis catalyst. Some attempts including the use of Lewis acids,^11−13^ ionic liquids, Brønsted/Lewis acids in combination with ionic liquids,^14^ Brønsted acidic ionic liquids,^15,16^ Brønsted acidic ionic liquids with metal salts as cocatalysts^17^ plus immobilized acidic ionic liquids^18,19^ are well-known.

**Figure 1:**
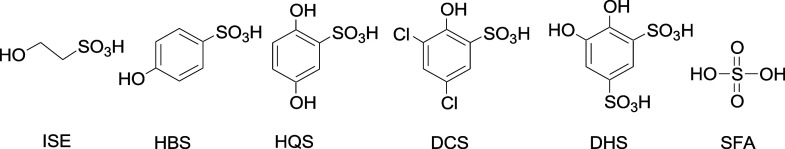
Acids used in comparison of cellulose hydrolysis catalytic activity study: isethionic acid (ISE), 4-hydroxybenzenesulfonic acid (HBS), hydroquinone sulfonic acid (HQS), 3,5-dichloro-2-hydroxybenzenesulfonic acid (DCS), 1,2-dihydroxybenzene-3,5-disulfonic acid (DHS), and sulfuric acid (SFA).

The −SO_3_H group catalyzed cellulose depolymerization is a widely researched topic in heterogeneous catalysis where −SO_3_H groups are generally immobilized on a solid surface such as carbon, graphene, silica, or polymeric support.^20−23^ However, the use of −SO_3_H group based catalysts in the homogeneous phase is rare, and in our previous work we have studied the catalytic activities of a series of alkyl/aryl sulfonic acids in water for the hydrolysis of sigmacell cellulose to reveal the superior activity of some hydrophobic aryl sulfonic acids when compared to sulfuric acid. For instance, high molecular weight cellulose hydrolyzed in dilute aqueous solutions of *p*-toluenesulfonic, 2-naphthalenesulfonic, and 4-biphenylsulfonic acid mediums produced total reducing sugar (TRS) yields of 28.0, 25.4, and 30.3%, respectively, in comparison to 21.7% TRS produced in aqueous sulfuric acid under similar conditions. As an extension of this work we have recently studied a series of hydroxy sulfonic acids shown in Figure 1 as simple cellulase enzyme model catalysts and compared their cellulose hydrolysis activities with dilute aq. sulfuric acid.^24^ In these experiments, the two-carbon hydroxy acid isethionic acid showed the highest catalytic activity, producing 62.7% TRS yield at 180 °C, 4 h. In the next section of this study, Density Functional Theory (DFT) calculations were performed on the cellulose model compound D-cellobiose to correlate the experimental results with the structure of the catalysts. Furthermore, binding energies of D-cellobiose–hydroxy sulfonic acid pairs and the distance between hydroxy-sulfonic acid −SO_3_H acidic H and glycosidic oxygen were evaluated. Interestingly, the −SO_3_H acidic H to glycosidic oxygen distance was identified as the significant parameter correlated to the hydroxy-sulfonic acid catalytic activity. In the set of hydroxy sulfonic acids studied, isethionic acid showed the highest cellulose hydrolysis catalytic activity and in the DFT study displayed the shortest −SO_3_H to glycosidic oxygen distance of 1.744 Å.

**Figure 2:**
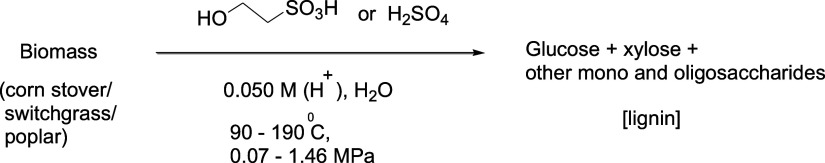
Hydrolysis of lignocellulosic biomass varieties corn stover, switchgrass, and poplar using dilute aqueous isethionic or sulfuric acid as catalysts.

Since we have identified isethionic acid as the most active among the set in Figure 1 and a better catalyst than sulfuric acid for hydrolysis of moderately high molecular weight cellulose, our next step was to test isethionic acid in hydrolysis of real biomass samples. During this stage 0.050 mol H^+^/L isethionic and sulfuric acid hydrolysis of three different lignocellulosic biomass varieties, switchgrass, corn stover, and poplar, were compared at 90–190 °C temperature range as shown in Figure 2, and the results of these experiments are presented in this publication. The weight percentage compositions of biomass varieties switchgrass, corn stover, and poplar are shown in Table 1. Even though isethionic acid can be easily prepared by condensation of inexpensive SO_2_, NaOH, and ethylene oxide; this simple sulfonic acid was hardly ever used as a catalyst in a chemical transformation.^25^ Apart from our previous experiments,^24^ Ishida and co-workers tested isethionic acid immobilized on a carbon surface as a solid acid catalysts for the hydrolysis of cellulose to glucose in 1-butyl-3-methyl-imidazolium chloride under microwave irradiation at 120 °C, under a N_2_ atmosphere.^26^ However, the experiment of Ishida and co-workers resulted in catalytic activities less than that of sulfuric acid immobilized carbon.^26^ This is probably due to the fact that when isethionic acid is immobilized on a carbon surface the bifunctional molecule is tethered to the surface via ether or ester link and is no longer an alcohol capable of hydrogen bonding with the polysaccharide, as presented in their work.

**Table 1:** Weight Compositions of Corn Stover,^31^ Switchgrass,^32^ and Poplar^33^

component	corn stover (weight %)	switchgrass (weight %)	poplar (weight %)
cellulose	37.4	43.8	43.2
hemicellulose	27.6	28.8	26.6
lignin	18.0	9.2	21.3
protein	3.1	3.9	
starch		1.0	
fat	4.7	0.9	
water-soluble	1.1	2.2	1.3
moisture	3.3	5.2	6.1
ash	4.8	5.0	1.5

## 2 Experimental Section

### 2.1 Materials and Instrumentation

Corn stover, switchgrass, and poplar used in this work are gift samples from the National Renewable Energy Laboratory, Boulder, Co, USA. These biomass samples were pulverized in a Bell-Art Micro Mill II grinder to obtain a homogeneous powder and sieved using an Aldrich mini-sieve (Z 675415, size 25; particle size, 0.7 mm). The powders were dried in an oven at 50 °C for over 24 h to a constant weight and stored in sealed bottles until used for experiments. 3,4-Dinitrosalicylic acid (>99%), sodium phosphate, conc. sulfuric acid (98%), and hydrochloric acid (37%), *o*-dianisidine (>99%), glucose oxidase (181.3 units/mg), and peroxidase (59 units/mg) were from Aldrich Chemical Co, St. Louis, MO. Sodium isethionate (98.0+%) is from Thermo Fischer Scientific, Waltham, MA. Stainless steel solvothermal reaction kettles with 25 mL capacity Teflon inner sleeves were used for biomass hydrolysis experiments. These reactors were purchased from Lonsino Medical Products Co. Ltd. Jingsu, China. Reactors were heated in a Precision Scientific model-28 oven with accuracy ±1 °C. Total reducing sugars (TRS) in biomass hydrolyzates were measured by 3,4-dinitrosalicylic acid (DNS) assay.^27,28^d-Glucose in hydrolyzates were measured using glucose oxidase-peroxidase assay.^29^ Preparation of DNS reagent and glucose oxidase-peroxidase assay reagent is included in the Supporting Information. These colorimetric assays were carried out using a GENESYS 150 UV–vis spectrophotometer from Thermo Scientific and Fisherbrand 1.0 cm polystyrene cuvettes from Thermo Fischer Scientific, Pittsburgh, PA. Attenuated total reflection infrared (ATR-IR) spectra of biomass and lignin residue samples in the 500–4000 cm^–1^ range were recorded using a Smiths IdentifyIR spectrometer (Danbury, CT, USA).

### 2.2 General Procedure for Hydrolysis of Biomass Samples Using Aqueous Isethionic and Sulfuric Acid Solutions

Isethionic and sulfuric acid stock solutions were prepared by dissolving appropriate amounts of these acids in deionized water to obtain an acid concentration of 0.050 mol H^+^/L in each solution. Furthermore, concentrations of these acid solutions were confirmed by titration with a standardized 0.050 M aqueous NaOH using phenolphthalein as the indicator. Biomass sample (0. 0500 g) was suspended in 2.00 mL of 0.050 mol H^+^/L aqueous acid solution in a high-pressure reactor. The reactor was tightly closed and heated in a thermostated oven (90 to 190 °C) for 4.0 h. Then the reactor was withdrawn from the oven and immediately cooled in an ice water bath for 30 min. Cooled reactor was opened and the contents were transferred to a 15 mL glass centrifuge tube, diluted to 10.0 mL with deionized water, neutralized with 0.10 M aqueous sodium hydroxide, then centrifuged at 3500 rpm for 10 min to precipitate the solids. The clear supernatant liquid was decanted and total reducing sugars and d-glucose in hydrolyzates were measured as shown in the procedures below.

### 2.3 Analysis of Total Reducing Sugar (TRS)

**Figure 3:**
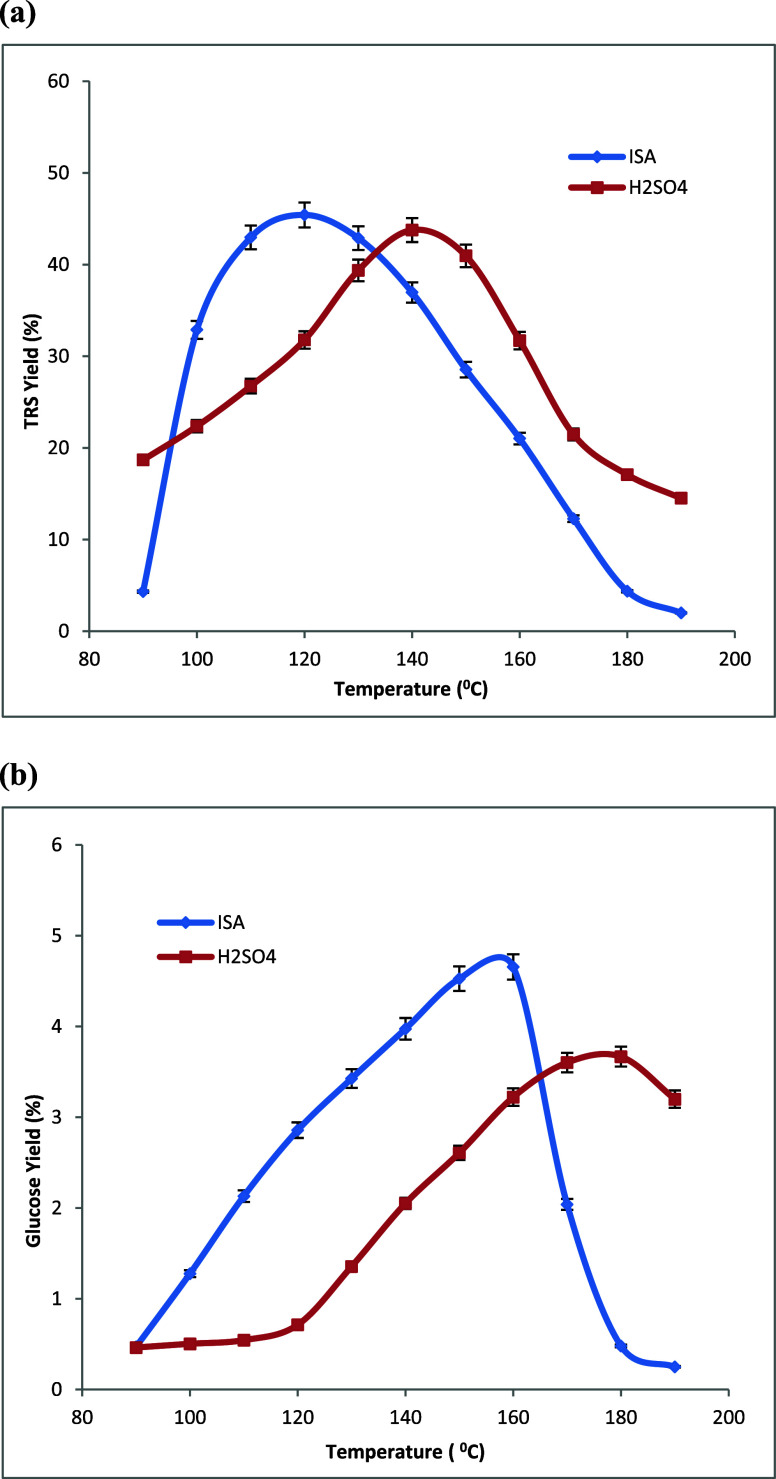
Changes in the yields of (a) total reducing sugars (TRS) and (b) d-glucose produced during the hydrolysis of corn stover in dilute aq. isethionic acid (ISA) and H_2_SO_4_ at different temperatures. All acid solutions: 0.050 mol H^+^/L, time = 4.0 h, and 0.050 g of corn stover in 2.00 mL of aq acid in all experiments. Averages of duplicate experiments.

**Figure 4:**
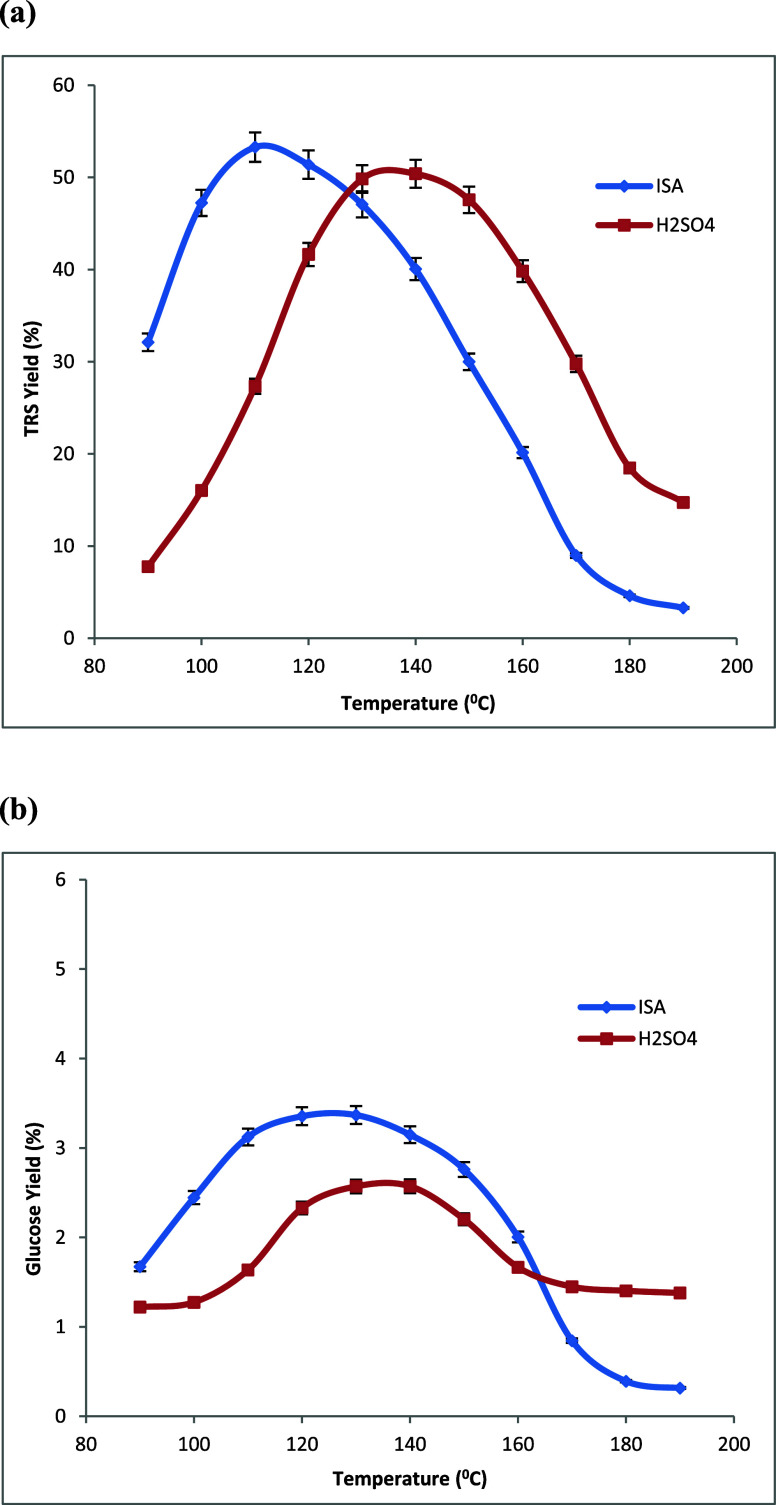
Changes in the yields of (a) total reducing sugars (TRS) and (b) d-glucose produced during the hydrolysis of switchgrass in dilute aq. isethionic acid (ISA) and H_2_SO_4_ at different temperatures. All acid solutions: 0.050 mol H^+^/L, time = 4.0 h, and 0.050 g of switchgrass in 2.00 mL of aq acid in all experiments. Averages of duplicate experiments.

**Figure 5:**
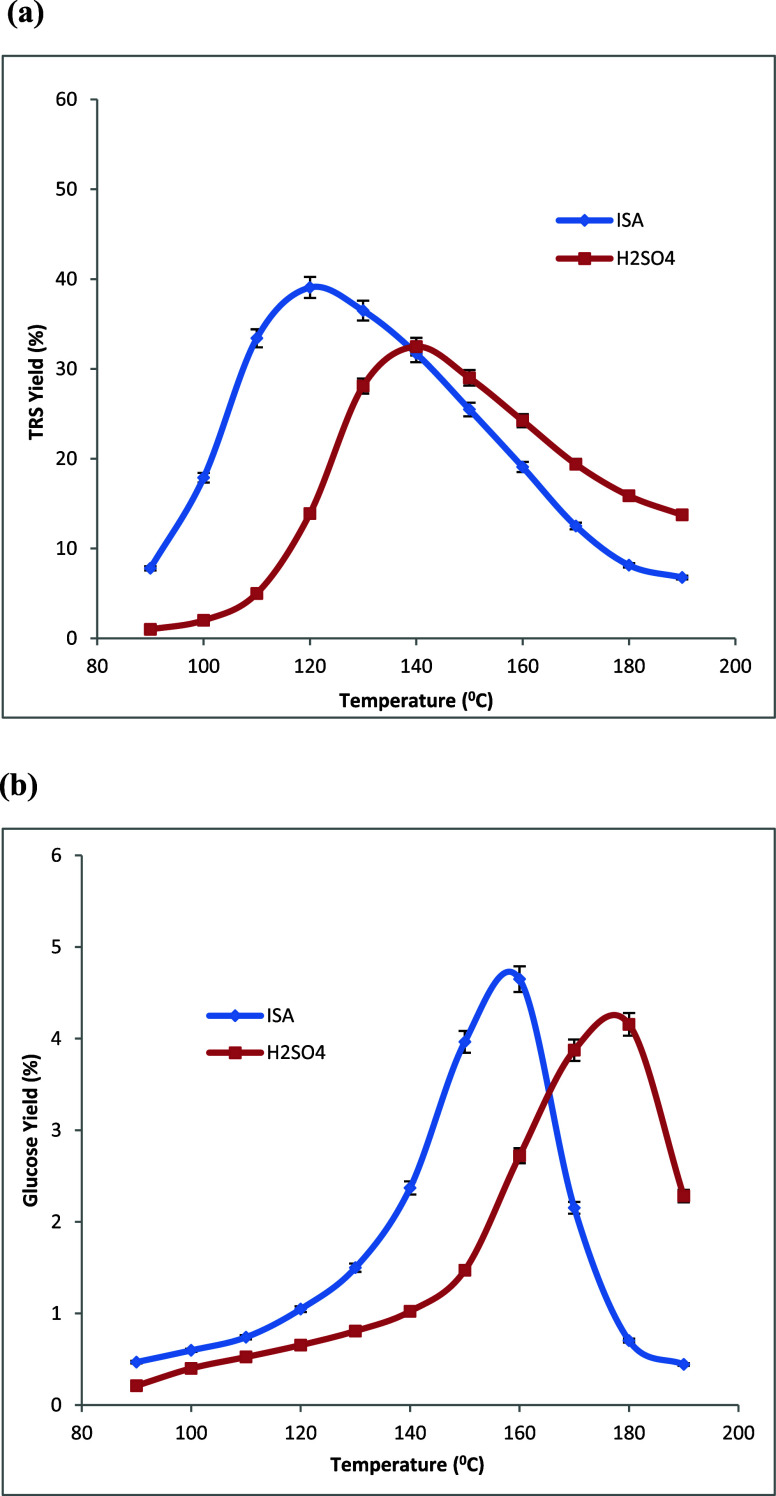
Changes in the yields of (a) total reducing sugars (TRS) and (b) d-glucose produced during the hydrolysis of poplar in dilute aq. isethionic acid (ISA) and H_2_SO_4_ at different temperatures. All acid solutions: 0.050 mol H^+^/L, time = 4.0 h, and 0.050 g of poplar in 2.00 mL of aqueous acid in all experiments. Averages of duplicate experiments.

A 1.00 mL portion of the hydrolyzate was transferred to a 20 mL glass vial and diluted with 2.50 mL of deionized water, and 0.50 mL of DNS reagent was added. The mixture was incubated in a water bath maintained at 90 °C for 5 min to develop a red-orange color. A reagent blank was prepared by mixing 3.50 mL of deionized water and 0.50 mL of DNS reagent and was heated the same way as the samples. The absorbance of the test sample at 540 nm was measured against the reagent blank using 1.0 cm polystyrene cuvets. The TRS concentrations in test solution were calculated by using a standard curve prepared by using a series of d-glucose standards. Changes in the TRS yields during the hydrolysis of corn stover, switch grass, and poplar in dilute aq. isethionic acid (ISA) and H_2_SO_4_ at different temperatures are shown in Figures 3a, 4a, and 5a, respectively.

### 2.4 Analysis of d-Glucose

A 1.00 mL portion of the hydrolyzate was transferred to a 20 mL glass vial and diluted with 1.00 mL of deionized water. The reaction was initiated at zero time by adding 2.00 mL of glucose oxidase-peroxidase reagent, mixing well, and incubating at 37 °C for 30 min in a water bath. At the end of the period, 2.00 mL of 6 M hydrochloric acid was added, resulting in a pink solution. 2.00 mL of deionized water with 2.00 mL of oxidase-peroxidase reagent was mixed to prepare the reagent blank and was treated the same way as the test sample. Then the absorbance of the test sample at 540 nm was immediately measured against the reagent blank using 1.0 cm polystyrene cuvets. The d-glucose concentrations in the test solution were calculated using a standard curve prepared using a series of d-glucose standards. Changes in the total d-glucose yields during the hydrolysis of corn stover, switch grass, and poplar in dilute aq. isethionic acid (ISA) and H_2_SO_4_ at different temperatures are shown in Figures 3b, 4b, and 5b, respectively.

### 2.5 FT-IR and Crystallinity Study of Biomass and Hydrolysis Residue

**Figure 6:**
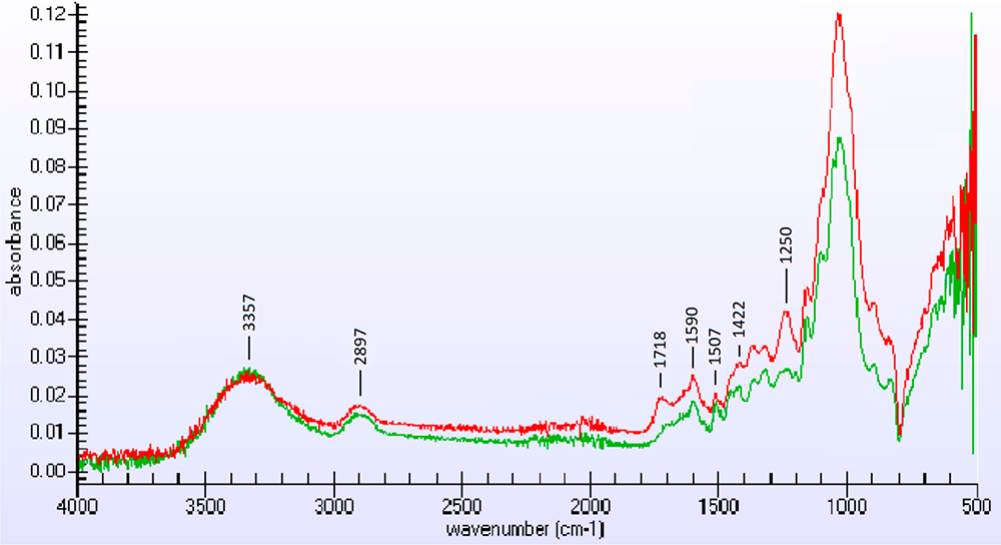
Representative FT-IR spectra of corn stover (red line) and lignin residue (green line) remaining after isethionic acid catalyzed hydrolysis of the polysaccharide fraction at 110 °C for 4.0 h.

Corn stover, switch grass, and poplar samples were dried in an oven at 50 °C, for 24 h and stored in a desiccator until the FT-IR spectra were recorded. Similarly, biomass hydrolysis residues from isethionic acid catalyzed hydrolysis experiments producing the highest TRS yields were dried and stored in a desiccator until the FT-IR spectra were recorded. Attenuated total reflection infrared (ATR-IR) spectra of these biomass and lignin residue samples were recorded in the 500–4000 cm^–1^ range. Representative FT-IR spectra of corn stover and lignin residue remaining after isethionic acid catalyzed hydrolysis of polysaccharide fraction at 110 °C for 4.0 h are shown in Figure 6. Crystallinity Index (CI) of biomass samples were calculated before and after isethionic acid hydrolysis using the formula^30^
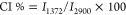
where *I*_1372_ and *I*_2900_ are IR absorption intensities at 1372 and 2900 cm^–1^ bands, respectively.

## 3 Results and Discussion

### 3.1 Isethionic and Sulfuric Acid Catalyzed Hydrolysis of Biomass

The catalytic activities of 0.050 mol of H^+^/L isethionic and sulfuric acid solutions were tested on three common biomass varieties in an attempt to improve the classical aqueous acid hydrolysis process. Isethionic acid was chosen for this study from a series of aliphatic and aromatic hydroxy acids, based on our previous study, where we compared five hydroxy sulfonic acids shown in Figure 1 for the hydrolysis of cellulose.^24^ As discussed in the introduction, isethionic acid showed the highest catalytic activity among catalysts in Figure 1, encouraging us to test isethionic acid on untreated real biomass samples. Corn stover, switch grass, and poplar hydrolysis experiments were carried out in aqueous acid solutions of the same strength: 0.050 mol H^+^/L and time = 4.0 h. All experiments were carried out in duplicate as described in the Experimental Section. The variation of TRS and glucose readings is approximately 3%.

Three biomass varieties, corn stover, switchgrass, and poplar, were chosen for the study due to their high combined cellulose and hemicellulose percentages of 65.0, 72.6, and 69.8%, respectively, as shown in Table 1. The maximum TRS yields of 50 and 54% with H_2_SO_4_ and ISA catalysts were achieved with switch grass biomass sample containing the highest polysaccharide content as expected. In addition, switch grass has the lowest lignin content of 9.2% out of all three biomass samples. The lowest TRS yields of 32 and 39% under H_2_SO_4_ and ISA catalysts were observed from poplar biomass; this outcome is probably due to the high 21.3% lignin content in poplar in comparison to other two biomass varieties. Since FT-IR studies on biomass residues also indicate the resilience of the lignin fraction during the acid hydrolysis, high lignin content is a major obstacle for both H_2_SO_4_ and ISA catalyzed hydrolysis of biomass. The d-glucose in hydrolysates is from the hydrolysis of cellulose fraction. The cellulose to hemicellulose ratios for CS, SG, and PL are 1.36, 1.52, and 1.62, respectively. The highest d-glucose yields of 4.2 and 4.7% were observed under H_2_SO_4_ and ISA catalysis, respectively, while using poplar biomass; and this is likely due to the fact that poplar has the highest cellulose to hemicellulose ratio in comparison to the other two biomass varieties studied.

As demonstrated in this study, this simple two carbon hydroxy acid is clearly a better catalyst than sulfuric acid. Isethionic acid consistently produced higher maximum TRS and glucose yields from CS, SG, and PL than conventional sulfuric acid of the same acid strength, as shown in Figures 3–5 and as summarized in Table 2. More significantly, with the use of isethionic acid instead of sulfuric acid, maximum TRS and glucose percentage yields could be achieved at considerably lower temperatures. The maximum TRS % yield peak positions for isethionic acid are 25, 24, and 21 °C lower than those for sulfuric acid when tested with CS, SG, and PL samples, respectively (Table 2). Similarly, the maximum glucose % yield peak positions for isethionic acid are 20, 13, and 20 °C lower than those for sulfuric acid when tested using CS, SG, and PL samples, respectively (Table 2).

**Table 2:** Data Summary from Figures 3–5^a^

biomass	H_2_SO_4_ TRS_max_ yield (%), peak (°C)	ISA TRS_max_ yield (%), peak (°C)	H_2_SO–ISA TRS peak shift (°C)	H_2_SO_4_ GLU_max_ yield (%), peak (°C)	ISA GLU_max_ yield (%), peak (°C)	H_2_SO_4_–ISA GLU peak shift (°C)
CS	43, 143	45, 118	25	3.7, 177	4.7, 157	20
SG	50, 134	54, 110	24	2.5, 137	3.4, 124	13
PL	32, 141	39, 120	21	4.2, 178	4.7, 158	20

a The maximum TRS and glucose % yields and peak positions (°C) data were acquired during the hydrolysis of three biomass varieties in 0.050 mol H^+^/L sulfuric and isethionic acid (ISA) solutions at 90–190 °C. CS, corn stover; SG, switchgrass; PL, poplar.

The maximum TRS and glucose yields in all three biomass varieties showed improvement by changing the acid catalyst from H_2_SO_4_ to ISA owing to the better catalytic activity of ISA. The most significant 32 to 39% enhancement in maximum TRS yield was found in poplar biomass with relatively high lignin content, showing an advantage of ISA over sulfuric acid. The higher TRS and glucose yields as well as lowering of the maximum yield temperatures on using ISA in comparison to sulfuric acid can be explained as a result of interactions between the hydroxyl group in ISA with hydroxyl groups of polysaccharides. Hydrogen bonding and other dipolar interactions between the ISA hydroxyl group and polysaccharide −OH groups can help to bind the catalyst on the polysaccharide surface, penetrate into cellulose and hemicellulose polymers, approach the glycosidic oxygens in cellulose and hemicellulose, then transfer the acidic H^+^ of ISA-SO_3_H group to the glycosidic oxygen for the hydrolysis. Whereas, sulfuric acid has no neighboring hydroxyl group to bind on the polysaccharide surface through dipolar interactions and promote the catalytic activity. Moreover, this neighboring hydroxyl group effect may lower the activation energy of the reaction, allowing hydrolysis reactions to reach peak TRS and glucose values at much lower temperatures and achieve higher yields as shown in Table 2. Furthermore, isethionic acid can be easily prepared by condensation of inexpensive SO_2_, NaOH, and ethylene oxide making it an attractive alternative to sulfuric acid.^25^

The sweeping lowering of reaction temperature to achieve good TRS and glucose yields in dilute aqueous isethionic acid is a considerable savings in energy in comparison to the standard sulfuric acid process. In addition, higher maximum TRS and glucose yields were also realized by using isethionic acid in comparison to sulfuric acid of the same acid concentration under similar conditions.

### 3.2 FT-IR and Crystallinity Index Analysis

We have analyzed the FT-IR spectra of the starting untreated biomass samples and residue remaining after isethionic acid catalyzed hydrolysis of the polysaccharide fraction in experiments producing the highest TRS yields, in an attempt to understand the hydrolysis process. The representative FT-IR spectra of starting corn stover (red) and residue (green) after isethionic acid catalyzed hydrolysis of the polysaccharide fraction at 110 °C for 4.0 h are shown in Figure 6. The characteristic lignin aromatic ring skeletal vibration absorption peaks at 1422, 1507, and 1159 cm^–1^ in corn stover (red) remains unchanged as shown in the IR spectrum of residue (green),^34,35^ whereas the strong primary alcohol C–O stretching absorption at 1250 cm^–1^ in raw biomass is diminished due to hydrolysis of cellulose and hemicellulose polymers in corn stover. Furthermore, weak C=O stretching absorption due to small amount of acetate functional groups in corn stover also disappeared due to the acid hydrolysis of these groups. The other two biomass varieties, switchgrass and poplar also showed similar changes in comparison of FT-IR spectra of raw biomass and hydrolysis residues, indicating that dilute aq. isethionic acid catalyzes the hydrolysis of cellulose and hemicellulose fractions, while lignin remains unchanged during the process. The crystallinity indexes (CIs) of starting corn stover and residue after isethionic acid catalyzed hydrolysis of polysaccharide fraction were calculated as 1.80 and 1.52, respectively, using the formula described in the experimental section. This experiment also demonstrates the effect of hydroxy sulfonic acid catalyst penetration of biomass, disrupting hydrogen bonding net work and hydrolyzing the polysaccharide fraction. Similarly, other biomass varieties also showed similar reduction in CIs after aqueous isethionic acid hydrolysis reactions.

## 4 Conclusion

The catalytic activity of isethionic acid was compared with the classical acid catalyst, sulfuric acid, for hydrolysis of polysaccharides in three different biomass samples: corn stover, switchgrass, and poplar. Total reducing sugar and glucose yields produced under different reaction temperatures were monitored for a series of hydrolysis experiments carried out with constant acid strength under a fixed reaction time. Isethionic showed higher maximum TRS and glucose yields in all three biomass samples at much lower temperatures in comparison to those of sulfuric acid catalyzed hydrolysis experiments. In comparison to sulfuric acid, the use of isethionic acid resulted in an average lowering of the maximum TRS and glucose yield temperatures by 23 and 18 °C, respectively. This significant reaction temperature reduction is most likely due to the lowering of the activation energy in the cellulose and hemicellulose depolymerization reaction through hydrogen-bonding-type intermolecular interactions between the isethionic acid hydroxyl group and the polysaccharide hydroxyl groups. Furthermore, an FT-IR analysis of the residue and comparison with the initial biomass revealed that the lignin fraction in biomass is not affected by the dilute aqueous isethionic acid catalyzed hydrolysis process. In conclusion, we have shown that dilute aqueous isethionic acid is a superior acid catalyst to classical sulfuric acid in the hydrolysis of untreated biomass to glucose and reducing sugars and their oligomers under relatively mild hydrothermal conditions.

## Supporting Information Available

Preparations of DNS and glucose oxidase–peroxidase assay reagents (PDF)
