# Supplementary material for: A Comparison of Dilute Aqueous Isethionic Acid and Sulfuric Acid in Hydrolysis of Three Different Untreated Lignocellulosic Biomass Varieties
Source: Ind Eng Chem Res. 2023 Nov 2;62(46):20037–43. doi: 10.1021/acs.iecr.3c02314 (PMC10863032; doi:10.1021/acs.iecr.3c02314)
Supplement: Supplementary file 1 — ie3c02314_si_001.pdf [file ie3c02314_si_001.pdf]

A Comparison of Dilute Aqueous Isethionic acid and Sulfuric acid in Hydrolysis of Three Different Untreated Lignocellulosic Biomass Varieties

Ananda S. Amarasekara[a,b]*, Victor C. Nwankwo[a, #]

[a]Department of Chemistry
Prairie View A&M University, 700 University Drive, Prairie View, Texas 77446, USA

[b]Center for Energy and Environmental Sustainability
Prairie View A&M University, 700 University Drive, Prairie View, Texas 77446, USA

*Email: asamarasekara@pvamu.edu

Present address:
[#]V.C.N.: Texas A&M University, College Station, Texas 77843, USA

---

(1). Preparation of 3,4-Dinitrosalicylic acid reagent

10.92 g of potassium tartarate and 0.378 g of 3,4-dinitrosalicylic acid were dissolved in 30.0 mL of hot water at 50 ºC. A second solution was prepared by dissolving 0.30 g phenol and 0.30 g sodium sulfite in 15.70 mL of 2 M aqueous sodium hydroxide. Then the two solutions were mixed, stirred until a homogeneous solution is formed, cooled to room temperature and diluted to give 60 mL of DNS reagent. The reagent stored in a brown glass bottle is stable up to 6 months in the refrigerator.

(2). Preparation of glucose oxidase - peroxidase assay reagent

0.020 g of *o*-dianisidine was dissolved in 1.0 mL of methanol and mixed with 79.0 mL of 0.1 M sodium phosphate buffer with pH 6.5 to prepare the buffered - *o*-dianisidine solution. The glucose analysis reagent was prepared by dissolving 0.0055 g of glucose oxidase (181.3 units/mg) and 0.0035 g of peroxidase (59 units/mg) enzymes in this buffered - *o*-dianisidine solution. This reagent was kept in a brown glass bottle, frozen at -20 °C, and stable for up to 6 months.
